# Antidepressant drug-specific prediction of depression treatment outcomes from genetic and clinical variables

**DOI:** 10.1038/s41598-018-23584-z

**Published:** 2018-04-03

**Authors:** Raquel Iniesta, Karen Hodgson, Daniel Stahl, Karim Malki, Wolfgang Maier, Marcella Rietschel, Ole Mors, Joanna Hauser, Neven Henigsberg, Mojca Zvezdana Dernovsek, Daniel Souery, Richard Dobson, Katherine J. Aitchison, Anne Farmer, Peter McGuffin, Cathryn M. Lewis, Rudolf Uher

**Affiliations:** 1 Biostatistics and Health Informatics Department. Institute of Psychiatry, Psychology and Neuroscience, Kings College London. 16 De Crespigny Park, London, SE5 8AF UK; 2 Social, Genetic and Developmental Psychiatry Centre, Institute of Psychiatry, Psychology and Neuroscience, King’s College London, 16 De Crespigny Park, Denmark Hill, London, SE5 8AF UK; 3 Department of Psychiatry, University of Bonn, Regina-Pacis-Weg 3, 53113 Bonn, Germany; 4 Central Institute of Mental Health, Division of Genetic Epidemiology in Psychiatry, Square J5, 68159 Mannheim, Germany; 5 Research Department P, Aarhus University Hospital, Norrebrogade 44, DK-8000 Aarhus C Risskov, Denmark; 6 Laboratory of Psychiatric Genetics, Department of Psychiatry, Poznan University of Medical Sciences, Collegium Maius, Fredry 10, 61-701 Poznań, Poland; 7 Croatian Institute for Brain Research, Medical School, University of Zagreb, 10 000 Zagreb, Salata 3 Croatia; 8 Vzgojni zavod Planina, Planina 211, 6232 Planina, Slovenina and Universitiy of Ljubljana, Medical Faculty, Vrazov trg 2, 1000 Ljubljana, Slovenia; 9 Laboratoire de Psychologie Médicale, Université Libre de Bruxelles and Psy Pluriel - Centre Européen de Psychologie Médicale, Av Jack Pastur 47a, 1180 Uccle, Belgium; 10 Department of Psychiatry and Medical Genetics, University of Alberta, 116 St and 85 Ave, Edmonton, AB T6G 2R3 Canada; 11 Dalhousie University Department of Psychiatry, 5909 Veterans’ Memorial Lane, Halifax, B3H 2E2 Nova Scotia Canada

## Abstract

Individuals with depression differ substantially in their response to treatment with antidepressants. Specific predictors explain only a small proportion of these differences. To meaningfully predict who will respond to which antidepressant, it may be necessary to combine multiple biomarkers and clinical variables. Using statistical learning on common genetic variants and clinical information in a training sample of 280 individuals randomly allocated to 12-week treatment with antidepressants escitalopram or nortriptyline, we derived models to predict remission with each antidepressant drug. We tested the reproducibility of each prediction in a validation set of 150 participants not used in model derivation. An elastic net logistic model based on eleven genetic and six clinical variables predicted remission with escitalopram in the validation dataset with area under the curve 0.77 (95%CI; 0.66-0.88; p = 0.004), explaining approximately 30% of variance in who achieves remission. A model derived from 20 genetic variables predicted remission with nortriptyline in the validation dataset with an area under the curve 0.77 (95%CI; 0.65-0.90; p < 0.001), explaining approximately 36% of variance in who achieves remission. The predictive models were antidepressant drug-specific. Validated drug-specific predictions suggest that a relatively small number of genetic and clinical variables can help select treatment between escitalopram and nortriptyline.

## Introduction

The reasons why some patients respond well to antidepressant medications but others do not benefit sufficiently from treatment are still poorly understood. Investigations of biologically related individuals from family studies^1^, non-related individuals from candidate gene studies^2^ and large-scale genome-wide association studies^3–7^ identified genetic contributions to treatment outcome. However, few associations with specific genetic variants were replicated and genetic polymorphisms explained only a small fraction of individual differences in antidepressant response. Other factors affecting the response to antidepressant drugs include the severity and type of depressive symptoms, prior exposure to adverse environment, and demographic factors. However, none of these provided differential prediction of alternative treatments outcomes with a clinically meaningful accuracy^8–12^.

The modest contributions of multiple clinical and genetic predictors suggest that a multivariate approach that combines genetic variants and clinical variables could improve the prediction of antidepressant treatment outcome. An initial application of statistical learning suggested that a combination of multiple clinical variables can improve the prediction over any single factor^12^. However, it is unknown whether a combination of genetic and clinical variables can improve the prediction of treatment outcomes further. Here, for the first time, we aim to maximise prediction of outcomes of treatment with alternative antidepressants using a combination of genetic, demographic and clinical measurements in patients with major depressive disorder. We report on a statistical learning analysis using more than 500,000 common genetic variants and 139 demographic and clinical variables to optimize the prediction of remission during treatment with a serotonergic or noradrenergic antidepressant.

## Results

### Prediction of remission during treatment with escitalopram

In the training dataset of escitalopram-treated participants, 17 variables were selected including HRSD total score and item Somatic Symptoms - General, the symptom dimensions of loss of interest-activity and appetite, BDI item sleep, SCAN item fatigability and 11 genetic markers (Tables 1 and 2).

**Table 1:** Variables selected and Odds ratio from elastic net logistic regression models estimated in the training data sets. OR: Odds Ratio.

Escitalopram N train = 143		Nortriptyline N train = 137	
Predictor	OR	Predictor	OR
Appetite (SCAN)	0.96	rs6794400	0.96
Changes sleep (BDI)	0.96	rs79693177	0.97
Somatic Symptoms (HRSD)	0.96	rs12874087	0.97
Interest-activity	0.97	rs2345113	0.97
HRSD total	0.97	rs17091959	0.97
Fatigability (SCAN)	0.98	rs10792321	0.97
rs1392611	0.97	rs199561596	0.97
rs10812099	0.97	rs144829540	0.97
rs1891943	0.98	rs149619279	0.98
rs151139256	0.98	rs34319049	0.98
rs11002001	0.98	rs151132095	0.98
rs62182022	0.99	rs37596	0.98
rs28373080	1.02	rs8053632	0.98
rs7757702	1.02	rs111685823	0.99
rs76557116	1.03	rs4279984	0.99
rs9557363	1.03	rs17057129	0.99
rs2704022	1.04	rs5889536	0.99
		rs34841556	1.01
		rs4773117	1.01
		rs8082631	1.02

OR: Odds ratio.

**Table 2:** Genetic markers included in elastic net models for predicting remission.

Gene	Marker	Chr:Position	Antidepressant	MAF	Allele
*SERP1* – Intron variant	rs6794400	3:150581092	Nortriptyline	0.057	A/C
*TMEM170A* – Intron variant	rs37596	16:75464422	Nortriptyline	0.32	A/C
*CFDP1* – Intron variant	rs8053632	16:75331042	Nortriptyline	0.23	C/T
*CCDC7* – Intron variant	rs111685823	10:32799271	Nortriptyline	0.0096	C/T
*TMEM2* – Intron variant	rs17057129	9:71698513	Nortriptyline	0.20	A/C
*SGCZ* – Intron variant	rs5889536	8:14517210	Nortriptyline	0.068	−/G
*SLC25A37* – Intron variant	rs34841556	8:23556091	Nortriptyline	0.446	−/CT
*ACCN1* - Intron variant	rs8082631	17:34064031	Nortriptyline	0.42	A/G
Intergenic	rs4773117	13:110066456	Nortriptyline	0.017	C/T
Intergenic	rs79693177	2:186199515	Nortriptyline	0.026	G/T
Intergenic	rs12874087	13:68211573	Nortriptyline	0.20	C/T
Intergenic	rs2345113	14:56675149	Nortriptyline	0.15	C/G/T
Intergenic	rs17091959	14:56691048	Nortriptyline	0.15	C/T
Intergenic	rs10792321	11:61979317	Nortriptyline	0.40	A/G
Intergenic	rs199561596	2:186510855	Nortriptyline		−/AT
Intergenic	rs144829540	2:186464172	Nortriptyline	0.15	A/G
Intergenic	rs149619279	9:122105909	Nortriptyline	0.08	A/G
Intergenic	rs34319049	20:38710108	Nortriptyline	0.03	C/T
Intergenic	rs151132095	2:186317904	Nortriptyline	0.15	C/T
Intergenic	rs4279984	11:37172240	Nortriptyline	0.094	C/T
*TMEM229B*	rs28373080	14:67506046	Escitalopram	0.49	C/T
*CDYL* – Intron variant	rs7757702	6:4940209	Escitalopram	0.45	A/T
*LOC105375673* – Intron variant	rs2704022	8:100728509	Escitalopram	0.42	A/C
Intergenic	rs1891943	13:53013037	Escitalopram	0.13	A/G
Intergenic	rs151139256	2:180139767	Escitalopram	0.026	−/T
Intergenic	rs11002001	10:52426412	Escitalopram	0.014	A/G
Intergenic	rs62182022	2:180060581	Escitalopram	0.15	C/T
Intergenic	rs76557116	13:100011900	Escitalopram	0.47	C/T
Intergenic	rs9557363	13:100032511	Escitalopram	0.47	C/T
Intergenic	rs1392611	4:45347307	Escitalopram	0.16	C/T
Intergenic	rs10812099	9:24797940	Escitalopram	0.23	A/T

MAF: Minor allele frequency.

**Figure 1:**
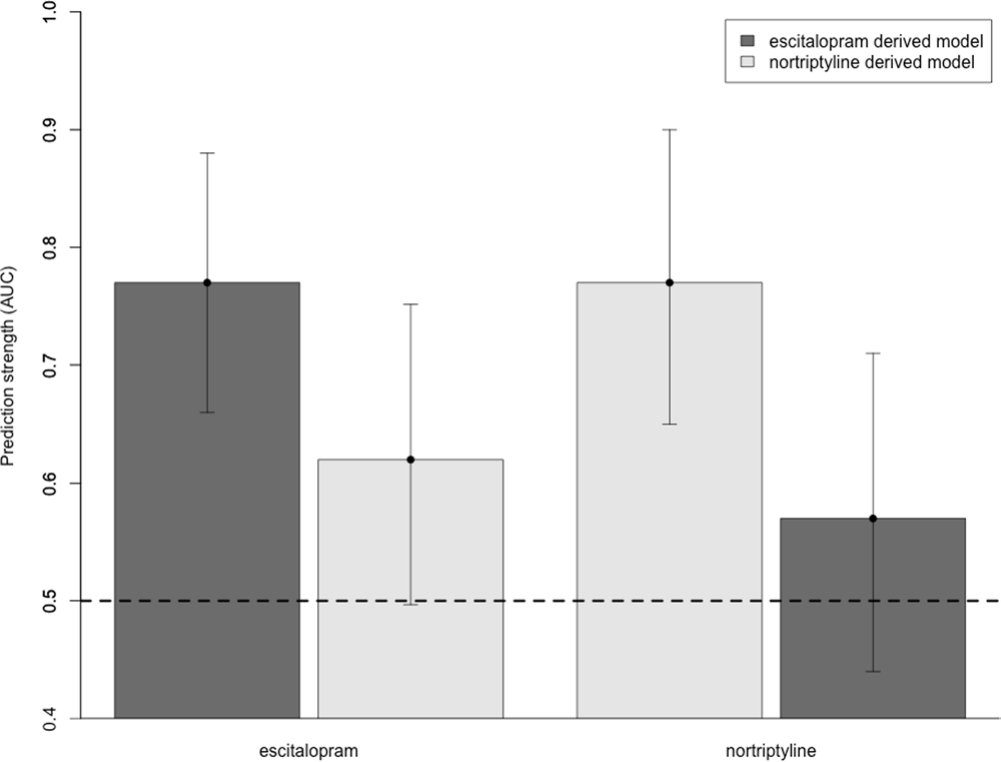
Remission prediction accuracy and specificity to antidepressant drug. AUC (Area Under the ROC curve) is shown for models trained and validated in samples treated with the same drug, and for models trained and validated in samples treated with different drug (cross-drug analysis). The horizontal dashed line marks the no discrimination level (AUC of 0.5). Vertical bars indicate a 95% confidence interval of the AUC estimate.

An elastic net logistic model based on these variables predicted remission in the training set with AUC 0.80 (95%CI [0.73–0.88]; p value < 0.001), sensitivity 0.71, specificity 0.77 and pseudo R^2^ 0.37. In external validation, the same model predicted remission in the non-overlapping validation dataset with AUC 0.77 (95%CI [0.66–0.88]; p value = 0.004), sensitivity 0.69, specificity 0.71 and pseudo R^2^ 0.30 (Fig. 1).

In cross-drug specificity analyses, the escitalopram-derived elastic net model predicted remission in nortriptyline-treated participants at chance level, with AUC 0.57 (95%CI [0.44–0.71]; p value = 0.29), sensitivity 0.46, specificity 0.67 and pseudo R^2^ 0.03, suggesting that prediction is drug-specific (Fig. 1).

### Prediction of remission during treatment with nortriptyline

In the training dataset of nortriptyline-treated participants, 20 variables were selected, all of them genetic variants (Tables 1 and 2). The elastic net logistic regression model derived from these 20 genetic variables predicted remission in the training set with AUC 0.83 (95%CI [0.76–0.91]; p value 0.003), sensitivity 0.7, specificity 0.83 and pseudo R^2^ 0.36. The model predicted remission in the non-overlapping validation dataset of nortriptyline-treated participants with an AUC 0.77 (95%CI [0.65–0.90]; p value < 0.001), sensitivity 0.68, specificity 0.87 and a pseudo R^2^ 0.36 (Fig. 1).

In cross-drug specificity analyses, the nortriptyline-derived elastic net model predicted remission in escitalopram-treated participants at chance level, with AUC 0.62 (95%CI [0.50–0.75]; p value = 0.062), sensitivity 0.29, specificity 0.52 and pseudo R^2^ 0.04, suggesting that prediction is drug-specific (Fig. 1).

## Discussion

The present results show that a combination of relatively few genetic and clinical variables can predict whether an individual with depression may reach remission with a specific antidepressant. The prediction models are parsimonious, based on only 17 and 20 variables, and the predictions are reproducible in non-overlapping validation datasets. These results demonstrate that a combination of genomic and clinical information in statistical learning framework has the potential to serve as a clinical decision support tool that may help select an antidepressant that an individual is more likely to benefit from.

The prediction was largely antidepressant-specific. The models predicted remission in validation sample treated with the same antidepressant, but not in samples treated with the other antidepressant. The drug-specificity makes the multivariate prediction more useful and applicable to clinical decision making. While the prediction of remission with escitalopram was driven by a combination of clinical and genetic variables, the achievement of remission with nortriptyline was predicted from genetic variants only. The clinical variables that contributed to the prediction of remission with escitalopram overlapped with previously reported predictors. Our model suggested that patients who had low levels of interest and activity, sleep problems, somatic symptoms and severe depression were less likely to reach remission, reflecting previously identified associations with symptom profiles^10,12,13^. For the prediction of response to nortriptyline, the procedure selected only genetic variables. The selection of only genetic variables in the nortriptyline-treated group suggests that the information predictive of nortriptyline response was better captured by genetic variables than the information predictive of response to escitalopram. The genetic variants selected into the prediction models were distinct from those identified in univariate genome-wide association studies^3–7^. For example, the genetic variants that predicted remission with nortriptyline in the multivariate model did not include the variant rs2500535 in *UST* that was previously identified as significantly associated with response to this antidepressant in the same dataset^7^. These results demonstrate that a statistical learning framework uses a multidimensional pool of predictors in a way that is partially distinct from traditional univariate approaches and has the potential to build novel prediction models that are relevant to clinical outcomes and robust in generalisation.

It is widely accepted that multiple genes/alleles are involved in determining response to antidepressants, some of which may not have been yet discovered. Interestingly, some of the genes containing variants that we reported as predictive of antidepressant treatment response have been recently identified as depression risk genes, as well as associated with bipolar disorder, schizophrenia and other brain diseases (Tables 1 and 2). For example, the *SGCZ* gene, part of the sarcoglycan complex, a group of six proteins which bridge the inner cytoskeleton and the extra-cellular matrix, has been recently reported to be associated with major depression, schizophrenia and bipolar disorder^14^, as well as with alcohol and nicotine co-dependence^15^, and Parkinson’s disease^16^. The consistent down-regulation in major depression patients in three independent samples suggested that *SCL25A37* may be used as a potential biomarker for major depression diagnosis^17^. This gene was also associated with fatigue^18^. The acid sensing ion channel (*ACCN1*) has been associated with response to lithium treatment in bipolar disorder^19^ and also associated with risk of autism^20^. The gene encoding the transmembrane protein 229 b has been associated with risk for Parkinson disease^21^ and with childhood obesity^22^. The gene *TMEM170A* encoding the transmembrane protein 170 A and the CFDP1, the craniofacial development protein 1, have been both associated with coronary risk disease^23^. The latter has been also associated with lung function^24^. Another variant identified in this work was located in the transmembrane protein 2 gene *TMEM2*, which has an essential role in coordination of myocardial and endocardial morphogenesis^25^. None of the selected genetic variations were located in genes previously associated with pharmacogenetics in depression treatment. However, it is a common finding in genomics that most predictive genetic variants are in locations other than the predicted candidate genes. This is responsible for the general failure of the candidate gene approach and it opens new ways for understanding pathogenesis and pharmacology. Surprising findings from genomic research in other disorders have open new ways of understanding and treating the disorders (e.g. the involvement of complement in macular degeneration, schizophrenia was previously unsuspected). Further functional characterization may provide potential targets for future therapeutic antidepressants.

The prediction was accurate enough to be clinically meaningful. Remission was predicted in validation data with an AUC of 0.77 in the escitalopram group and 0.77 in the nortriptyline group. Following the classification proposed by Hosmer & Lemeshow^26^ our models had “acceptable discrimination” (values of AUC of 0.7 or higher). The utility of biomarkers and prediction models in practice does not depend solely on their prediction accuracy, as reflected by the AUC, but also on clinical context, gravity of the predicted outcomes, cost and burden of the test. For example, a comparison among breast cancer prediction algorithms reported good performance for models having AUC’s below 0.7^27^. The fact that genetic and clinical variables used in the present model can be obtained with high accuracy and low-cost measurements that do not burden participants suggest that such models may be useful in practice.

Most of our previous work reporting on GENDEP applied analytical methods from the traditional inferential statistical framework, based on the assessment of *association* of a single clinical or genetic variant with treatment response in any given test. Association analysis aims to test the effects of specific factors on the response. This approach will highlight the predictive variable that has the strongest relationship with outcome on its own. In contrast, our current report aims to achieve an optimized *prediction* of outcome with the use of all available predictor variables, thus following a substantially different aim. Statistical learning can be used to build a model that will predict treatment outcome for new (unseen) cases, with clinical utility in practice. While explanatory power provides information about the strength of an underlying causal relationship, it does not imply its predictive power. By capturing underlying complex patterns and relationships, predictive modeling can suggest improvements to existing explanatory models^28^.

GENDEP has several strengths that make it suitable for prediction modeling. It is a randomised controlled trial that allows optimal comparison between treatments and the development of treatment-specific predictors^29,30^. The longitudinal study design of GENDEP allowed the follow-up of patients and the prospective assessment of symptom change, this being the most appropriate approach to establish cause-effect relations and avoid inconsistencies in data collection. The study was specifically designed to assess remission as the primary outcome, with patients being followed for 12 weeks. All patients had four or more depression severity measurements, with more than eighty percent of the sample having eight or more depression measurements, enough time to observe a clinical trend that could lead to clinical remission. However, interpretation of the present results has to take into account several limitations. First, while a wealth of information was available in the GENDEP dataset, not all relevant predictors were measured. For example, history of maltreatment in childhood has been shown to predict outcome of treatment with antidepressants^31^, but information on childhood maltreatment is not available in GENDEP. Second, since GENDEP only included individuals of white European ancestry without family history of bipolar disorder, the results may not generalize to individuals of other ethnicities or those with family history of bipolar disorder. Third, GENDEP only included two antidepressant drugs distinct in their mechanisms of action. Similar prediction of outcomes with other antidepressants, with neurostimulation and psychological treatments will require investigation in large and richly assessed samples of individuals treated with different modalities. Fourth, the GENDEP study was used as an exploratory dataset to build and test the predictive models. The clinical application of these models will require a comparison of outcomes between individuals whose treatment is selected according to a prediction model with those whose treatment is selected by chance or according to the judgement of the treating physician.

In conclusion, the present results demonstrate that a combination of a relatively small number of clinical and genetic variables can meaningfully and robustly predict remission with escitalopram and nortriptyline antidepressants among individuals with major depressive disorder. Statistical learning methods may be used to derive similar models for individuals treated with various antidepressants and other treatment modalities to map the opportunities for individualized indications for treatments.

The models are available online at https://gist.github.com/raqini/669c38a6329aa2231268770200519d64.

## Methods

### Participants

**Figure 2:**
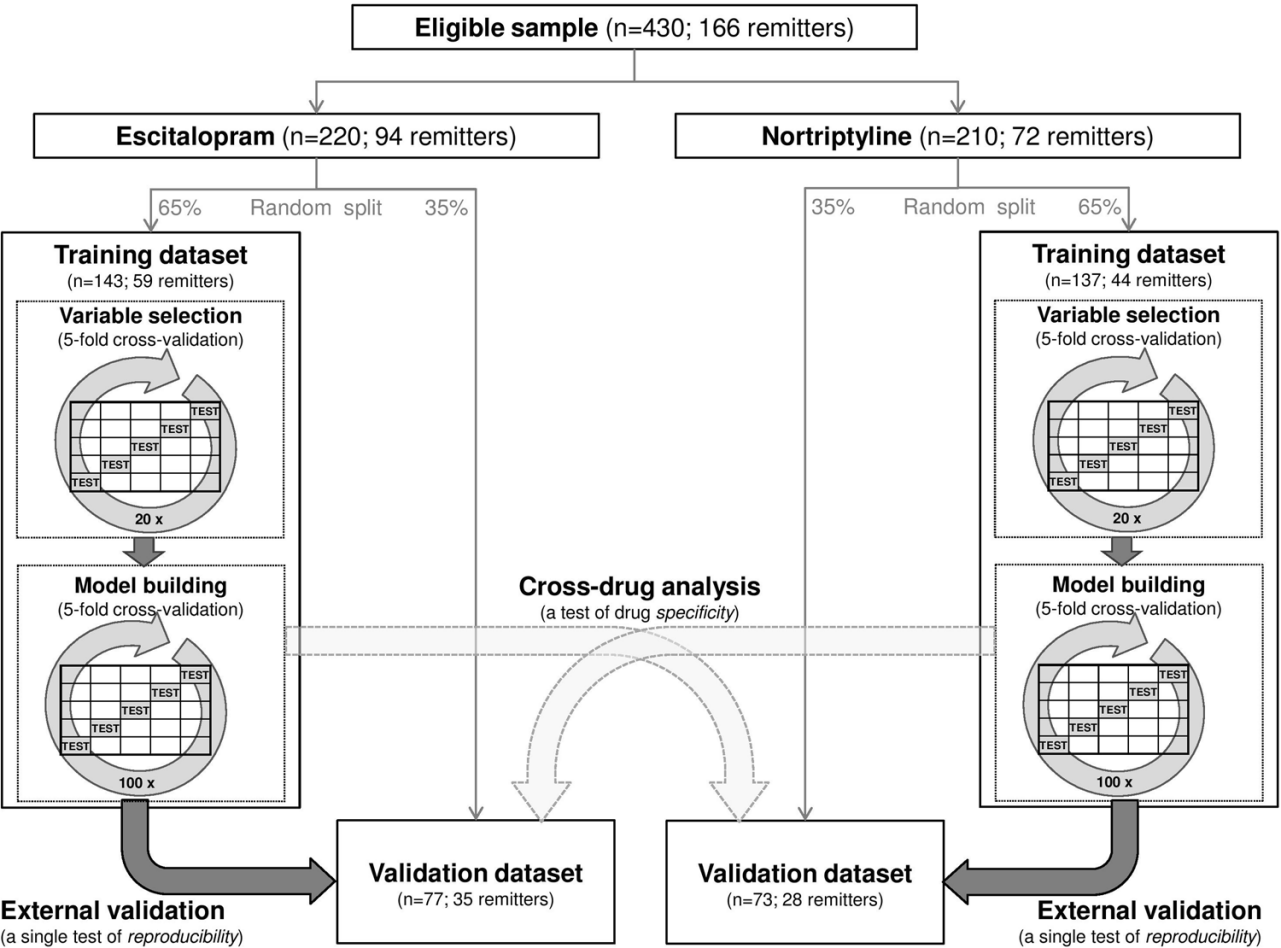
Flow diagram of sample division and analytic procedure used in variable selection, model derivation and validation.

We investigated treatment outcomes in 430 adults with major depressive disorder who were randomly allocated to receive either escitalopram, a selective serotonin reuptake inhibitor (SRI), or nortriptyline, a second-generation tricyclic antidepressant (TCA) that acts primarily as a norepinephrine reuptake inhibitor, and completed at least 4 weeks of treatment with the allocated antidepressant as part of the Genome-based Therapeutic Drugs for Depression (GENDEP)^7,32^. The two antidepressants were selected as representatives of different classes of antidepressants (SRI and TCA) that differ in their pharmacodynamics (serotonergic vs. primarily noradrenergic reuptake inhibition) and pharmacokinetics (distinct primary metabolizing enzymes). Genetic data for GENDEP participants were obtained in two phases. Firstly, 706 individuals were genotyped^7^. In a second phase, 105 more individuals were genotyped building a total sample of 811 individuals that were partially randomized to escitalopram and nortriptyline. Since our hypotheses concerned differential prediction and participants non-randomly allocated differed on some clinical characteristics^2^, we restricted the present analyses to the randomly allocated participants (n = 430). Randomisation has been shown to be crucial to avoid systematic confounding effect that might prevent predictive models from properly generalizing to other samples^33^. The participants were recruited from nine European centers and diagnosed with ICD-10/DSM-IV current depressive episode of at least moderate severity with the Schedules for Clinical Assessment in Neuropsychiatry (SCAN) interview^34^. Because of the genetic character of the study, the recruitment was restricted to individuals of white European parentage. Patients with personal or family history of bipolar disorder or schizophrenia and those with current substance dependence were excluded. They were treated for 12 weeks according to a protocol that guided dose adjustments according to response and tolerability, with 10 to 30 mg of escitalopram or 50 to 200 mg of nortriptyline daily. We randomly separated the participants into a training sample (65% of participants, a total of 280 patients) and a validation sample (the remaining 35%, a total of 150 patients) (Fig. 2) according to optimal percentage of split recommended to minimise predictive error^35^. The research ethic boards in all nine centers approved the study protocol. The ethics committee/institutional review board that approved GENDEP study in the lead center, King’s College London, was the *Joint South London and Maudsley and the Institute of Psychiatry NHS Research Ethics Committee* formed by Dr M Philpot (Co-Chair), Dr T Eaton (Co-Chair), Dr J Bearn, Professor T Craig, Professor A Farmer, Dr N Fear, Mr R Maddox, Mrs J Bostock, Dr V Kumari, Dr M Leese, Dr V Mouratoglou, Professor Sir Michael Rutter, Mr G Smith, Dr D Taylor, Dr U Ettinger, Mr J Watkins, Dr V Ng, Dr D Freeman and Dr T Joyce. All participants signed a written informed consent. All experiments were performed in accordance with relevant guidelines and regulations. The GENDEP study was registered at ISRCTN03693000 (www.controlled-trials.com) on 27^th^ September 2007. Participant characteristics are described in Supplementary Table S2.

### Outcome

The outcome was remission, defined as scoring 7 points or less on the 17-item Hamilton Rating Scale for Depression (HRSD)^36^ at the last available measurement after 4–12 weeks of treatment.

### Demographic and clinical predictors

All predictors were obtained at baseline, before participants received any study medication. Severity of depressive symptoms was assessed using three scales: the clinician-rated Montgomery–Åsberg Depression Rating Scale (MADRS)^37^, HRSD^36^ and the Beck Depression Inventory (BDI)^38^. Study interviewers collected information on gender, age, age at depression onset, body mass index (BMI), smoking (yes or not), years of education, marital status, occupation, and children (yes or not). The number of stressful life events in the 6 months previous to the interview was reported with the Brief List of threatening Events questionnaire (BLEQ)^39^. Medication information was recorded including the use of antidepressant at the time of recruitment, number of prior antidepressant trials and the types of antidepressants tried (SRI, tricyclic, dual, monoamine oxidase inhibitor or other antidepressants). Missing data were imputed by a bagged tree nonparametric method that allows inclusion of all cases without causing bias under a broad range of assumptions about missing data mechanisms^40^. Categorical data were rounded to plausible values after imputation^41^. In total, we included 139 clinical and demographic predictors (see Supplementary Table S1).

### Genotyping

DNA was extracted from blood samples collected in ethylenediaminetetraacetic acid^42^ and genotyped using the Illumina Human610-quad bead chip (Illumina, Inc., San Diego). This chip assays more than 610,000 single nucleotide polymorphisms (SNPs) and copy number variant markers selected to provide a comprehensive coverage across populations, and captures the majority of known common variation in the human genome, based on HapMap (release 23). Of the 550,337 SNPs with a minor allele frequency >0.01, a total of 539,391 (98%) were at least 99% complete and retained for analyses. The 430 participants presented no sex mismatches, no ambiguous genotypic sex and no outliers on heterozygosity. One individual in each of six pairs of related individuals (three first- and three second-degree pairs of relatives) was retained for further analyses. No population structure outliers were detected. The 430 individuals had a mean genotyping completeness of 99.82%. Using the IMPUTE v2 program^43^, we imputed missing SNPs data up to the 1000genomes (build 37). Quality control procedures and imputation are described in detail in Supplementary materials. Variants showing linkage disequilibrium (LD) over 0.8 were excluded from analysis. A total of 524871 common genetic variants were analysed.

### Data modeling

We randomly split the participants into mutually exclusive training dataset (65% of participants) and validation dataset (the remaining 35%; Fig. 2), a ratio that is optimal to minimise prediction error across a plausible range of achievable full dataset accuracy between 60% and 99%^35^. Within the training data set we performed 5-fold cross-validations to select informative variables and derive a statistical learning model to predict remission separately for escitalopram and for nortriptyline. The two resulting models (one for escitalopram and one for nortriptyline) were then externally validated in the validation dataset, a set of participants treated with the same drug that was not used in any way in the model derivation (Fig. 2). In addition, we probed drug-specificity of prediction by testing each predictive model in the validation dataset treated with the other drug. An additional analysis of the whole dataset of patients treated either with escitalopram or nortriptyline is reported in Supplementary materials.

### Variable selection in training data

In training data, we performed variable selection in 20 repetitions of a 5-fold cross-validation, 100 rounds in total. In each round, we left out one fifth of the training dataset and, in the remaining four-fifths of the training dataset, we estimated a Correlation-Adjusted T (CAT) score (i.e. a multivariate generalization of the standard univariate T-test statistic that takes the correlation among variables explicitly into account^44,45^ and the Local False Discovery Rate (LFDR) (i.e. the probability of a variable to be non-informative with regard to remission prediction given its CAT score) for each potential predictor. We retained predictors that had a LFDR smaller than 0.8 more times than not across the 100 rounds.

### Models development in training data

We used this set of variables to develop an elastic net logistic regression model in the training data set^46^. Elastic net model is a modified regression that allows to build multivariate models efficiently incorporating the correlation structure into the predictive accuracy calculation, whilst preventing the models from overfitting^47^. Parameters for the elastic net model need to be empirically determined. Following a procedure that optimizes the stability of results^48^, we carried out a 5-fold cross-validation with 100 repetitions to derive the parameters of a final predictive model.

### External validation of the models

For each antidepressant drug, we validated the final predictive model in the validation data set, an independent non-overlapping set of participants not used in any way in models derivation. We externally validated the prediction robustness and accuracy in the validation dataset of participants treated with the same drug. In addition, we evaluated drug-specificity of prediction by comparing same-drug (training and validation datasets treated with the same drug) with a cross-drug analysis (training and validation datasets treated with a different drug).

### Quantification of prediction accuracy

We indexed the accuracy of prediction with the Area Under the Curve (AUC) of a Receiver Operating Curve (ROC), sensitivity, specificity and Nagelkerke pseudo R^2^ coefficient. AUC^49^ can be interpreted as the probability that a classifier can identify (discriminate) a remitter when a remitter and a non-remitter cases are selected at random. The maximum value for the AUC is 1.0, thereby indicating a (theoretically) perfect discrimination (i.e., 100% sensitive, and 100% specific). An AUC value of 0.5 indicates no discriminative value (i.e., 50% sensitive and 50% specific). The Nagelkerke pseudo R^2^ approximates the proportion of outcome variance explained by the model.

### Statistical software used for analysis

We used caret^50^, sda^44,45^, glmnet^51^ and pROC^52^ libraries from R 3.2.3 statistical software^53^.

### Data availability statement

The data that support the findings of this study are available from the corresponding author on reasonable request. Data were used under license for the current study, and so are not publicly available.

## Electronic supplementary material

Supplementary material
