# Supplementary material for: Antidepressant drug-specific prediction of depression treatment outcomes from genetic and clinical variables
Source: Sci Rep. 2018 Apr 3;8:5530. doi: 10.1038/s41598-018-23584-z (PMC5882876; doi:10.1038/s41598-018-23584-z)
Supplement: Supplementary file 1 — Supplementary material [file 41598_2018_23584_MOESM1_ESM.pdf]

Antidepressant drug-specific prediction of depression treatment outcomes from genetic and clinical variables

Raquel Iniesta, Karen Hodgson, Daniel Stahl, Karim Malki, Wolfgang Maier, Marcella Rietschel, Ole Mors, Joanna Hauser, Neven Henigsberg, Mojca Zvezdana Dernovsek, Daniel Souery, Richard Dobson, Katherine J. Aitchison, Anne Farmer, Peter McGuffin, Cathryn M. Lewis, Rudolf Uher

Additional file 1: Supplementary materials

Quality control and population structure 2
- **References** 2

Analysis of the whole data set, including both drugs 3
- **Methods** 3
- **Results** 3
- **Discussion** 3
- **References** 4

Supplementary tables 5
- **Table S1: List of baseline predictors** 5
- **Table S2: Sample description** 10
- **Table S3: Information about markers included in models predicting remission and previously reported gene associations.** 11
- **Table S4: Variables selected and Odds ratio from elastic net logistic regression models estimated in the training data sets for the whole set of individuals, either treated with escitalopram or nortriptyline.** 12

Quality control and population structure

Quality control procedures were applied in PLINK (1), initially at the level of marker and then at the level of individual. Markers were retained if they had a minor allele frequency (MAF) of 0.01 or more as effects of rare markers would be uninterpretable with the present sample size. Markers were filtered for genotyping completeness of 99% so that all analyses were performed in a comparable set of individuals. Hardy-Weinberg Equilibrium (HWE) was not used as a filter as departures from HWE are expected in a case-only sample.

At the individual level, genotypes were first tested for sex mismatch with phenotypic data. Ambiguous genotypic sex and outliers on autosomal heterozygozity were investigated for exclusion as these may indicate sample contamination. Related individuals were ascertained through estimation of identity by descent (IBD) applied in PLINK to an LD- pruned dataset and one of each pair of first- or second-degree relatives (the one with less complete data) was excluded. Finally, genotyping completeness was assessed for each individual.

IMPUTE v2 program (2) was used to impute SNPs missing data to the 1000genomes (build 37). Given the minimal percentage of missingness, any missing value was completed following a best guess approach. Quality-control measures ensured only the most accurately imputed SNPs were used (info score filter of 0.1 and a genotype probability threshold of 0). We specified a Major Allele Frequency (MAF) of 0.005. Variants showing a linkage disequilibrium (LD) over 0.8 were excluded from analysis. A total of 524871 common genetic variants were analysed.

A genomic control lambda value was computed to assess false positive evidence of association due to genetic markers differing in genotype frequencies between subpopulations of remitters and non-remitters. As the inflation factor lambda was 0.9794552 (less than 1) no adjustment was necessary (3). Estimation was done using the GenABEL R package (4).

References

1. Purcell S, Neale B, Todd-Brown K, Thomas L, Ferreira MAR, Bender D, Maller J, Sklar P, de Bakker PIW, Daly MJ & Sham PC. PLINK: a toolset for whole-genome association and population-based linkage analysis. American Journal of Human Genetics. 2007; 81(3):559-575.
2. Howie B, Fuchsberger C, Stephens M, Marchini J, Abecasis GR. Fast and accurate genotype imputation in genome-wide association studies through pre-phasing. Nature genetics. 2012; 44:955-9.
3. Hinrichs A, Larkin E and Suarez B. Population Stratification and Patterns of Linkage Disequilibrium. Genet Epidemiol. 2009 ; 33(Suppl 1): S88–S92.
4. Aulchenko YS, Ripke S, Isaacs A, van Duijn CM. GenABEL: an R library for genome-wide association analysis. Bioinformatics. 2007; 23(10):1294-6.

Analysis of the whole data set, including both drugs

Methods

The aim of the analysis was to assess how demographic, clinical and genetic baseline information in combination predicted whether individuals achieved remission in the whole sample of patients treated either with escitalopram or nortriptyline. Same variables and steps of analysis used within every drug-specific group (and fully detailed in the methods section of the manuscript) were applied to the whole data set. To provide a completely independent test of each prediction model, we randomly split the data into mutually exclusive training dataset (65% of participants) and replication dataset (the remaining 35%). Sample sizes for training and test data sets were 280 and 150 respectively. The parameters for every model were estimated in the training dataset following a standard 5-fold cross validation approach. The predictive ability for the resulting model was then tested in the independent replication dataset, which was not used in any way in the model derivation.

Results

In the training dataset of participants treated with either escitalopram or nortriptyline, 12 variables were selected for the prediction of remission status. The selected predictors included the baseline total scores for HRSD, the symptom dimension of observed mood, loss of appetite and 9 genetic variants (Table S3, Table S4). The elastic net logistic regression model built from these 12 variables predicted remission in the replication dataset with an AUC of 0.69 (95%CI 0.61-0.76) and p value 0.017, sensitivity 0.68, specificity 0.69 and a pseudo R2 0.17 (Table S4).

Discussion

The collagen gene COL25A1 has implications in Alzheimer's disease (1) and has been associated with comorbid Antisocial Personality Disorder and Substance Dependence (2). c-Maf cooperates with Sox9 to activate the type II collagen gene (3). TBC1D8 gene has been shown to be predictive of risk of postpartum depression (4), as well as associated with osteoporosis (5) and identified as predictor of pancreatic cancer (6). The ITGB2 immunomodulatory gene and its protein CD18 was demonstrated in pruning neuronal synapses during brain development, with knockout mice for ITGB2 displaying deficits in synaptic connectivity along with several behavioural impairments (7-11). ITGB2 and its protein CD18 has been also associated with several conditions: papillary thyroid cancer (12), inflammatory mechanisms (13), vasculitis (14), Hirschsprung's disease (15), repair of the infarcted myocardium (16), obesity (17) and alcohol response (18).

References

1. Forsell C, Bjork BF, Lilius L, Axelman K, Fabre SF, Fratiglioni L*, et al.* (2010): Genetic association to the amyloid plaque associated protein gene COL25A1 in Alzheimer's disease. *Neurobiol Aging* 31:409-415
2. Li D, Zhao H, Kranzler HR, Oslin D, Anton RF, Farrer LA *et al.* (2012): Association of COL25A1 with comorbid antisocial personality disorder and substance dependence. *Biol Psychiatry* 71: 733–740.
3. Huang W, Lu N, Eberspaecher H, De Crombrugghe B (2002): A new long form of c-Maf cooperates with Sox9 to activate the type II collagen gene. *J Biol Chem* 277:50668-50675
4. Landsman A, Aidelman R, Smith Y, Boyko M, Greenberger C (2017): Distinctive gene expression profile in women with history of postpartum depression. *Genomics* 109:1–8
5. Hsu YH, Zillikens MC, Wilson SG, Farber CR, Demissie S, Soranzo N*, et al.* (2010): An integration of genome-wide association study and gene expression profiling to prioritize the discovery of novel susceptibility Loci for osteoporosis-related traits. *PLoS Genet* 6:e1000977.
6. Pezzilli R, Fabbri D, Imbrogno A (2011): Lymphocytes and pancreatic cancer: the effects of these cells on diagnosis and patient survival. *JOP* 12:209-210.
7. Zhan Y, Paolicelli RC, Sforazzini F, Weinhard L, Bolasco G, Pagani F*, et al.* (2014): Deficient neuron-microglia signaling results in impaired functional brain connectivity and social behavior. *Nat Neurosci* 17:400-406.
8. Stevens B, Allen NJ, Vazquez LE, Howell GR, Christopherson KS, Nouri N*, et al.* (2007): The classical complement cascade mediates CNS synapse elimination. *Cell* 131:1164-1178.
9. Schafer DP, Lehrman EK, Kautzman AG, Koyama R, Mardinly AR, Yamasaki R*, et al.* (2012): Microglia sculpt postnatal neural circuits in an activity and complement-dependent manner. *Neuron* 74:691-705.
10. Paolicelli RC, Bolasco G, Pagani F, Maggi L, Scianni M, Panzanelli P*, et al.* (2011): Synaptic pruning by microglia is necessary for normal brain development. *Science* 333:1456-1458.
11. Kettenmann H, Kirchhoff F, Verkhratsky A (2013): Microglia: new roles for the synaptic stripper. *Neuron* 77:10-18.
12. Eun YG, Kim SK, Chung JH, Kwon KH (2013): Association study of integrins beta 1 and beta 2 gene polymorphism and papillary thyroid cancer. *Am J Surg* 205:631-635.
13. Koch W, Bottiger C, Mehilli J, von Beckerath N, Neumann FJ, Schomig A, Kastrati A (2001): Association of a CD18 gene polymorphism with a reduced risk of restenosis after coronary stenting. *Am J Cardiol* 88:1120-1124.
14. Meller S, Jagiello P, Borgmann S, Fricke H, Epplen JT, Gencik M (2001): Novel SNPs in the CD18 gene validate the association with MPO-ANCA+ vasculitis. *Genes Immun* 2:269-272.
15. Moore SW, Sidler D, Zaahl MG (2008): The ITGB2 immunomodulatory gene (CD18), enterocolitis, and Hirschsprung's disease. *J Pediatr Surg* 43:1439-1444.
16. Wu Y, Ip JE, Huang J, Zhang L, Matsushita K, Liew CC*, et al.* (2006): Essential role of ICAM-1/CD18 in mediating EPC recruitment, angiogenesis, and repair to the infarcted myocardium. *Circ Res* 99:315-322.
17. Awaya T, Yokosaki Y, Yamane K, Usui H, Kohno N, Eboshida A (2008): Gene-environment association of an ITGB2 sequence variant with obesity in ethnic Japanese. *Obesity (Silver Spring)* 16:1463-1466.
18. Joslyn G, Ravindranathan A, Brush G, Schuckit M, White RL (2010): Human variation in alcohol response is influenced by variation in neuronal signaling genes. *Alcohol Clin Exp Res* 34:800-812.

Supplementary tables

Table S1: List of baseline predictors

List of baseline predictors			
**Demographic data and baseline severity**			
1	Age		
2	Age at onset		
3	Sex		
4	Smoking status (yes/no)		
5	Occupation (yes/no)		
6	Partner (yes/no)		
7	Years of education		
8	Children (yes/no)		
9	Body mass index		
10	Depression severity (MADRS)	Item: 1	Apparent Sadness
11		2	Reported Sadness
12		3	Inner Tension
13		4	Reduced Sleep
14		5	Reduced Appetite
15		6	Concentration Difficulties
16		7	Lassitude
17		8	Inability to Feel
18		9	Pessimistic Thoughts
19		10	Suicidal Thoughts
20			Total MADRS score
21	Depression severity (HRSD)	Item: 1	Depressed Mood
22		2	Feelings of Guilt
23		3	Suicide
24		4	Insomnia-Early
25		5	Insomnia-Middle
26		6	Insomnia-Late
27		7	Work and Activities
28		8	Retardation

29		9	Agitation
30		10	Anxiety – Psychic
31		11	Anxiety - Somatic
32		12	Somatic Symptoms - Gastrointestinal
33		13	Somatic Symptoms - General
34		14	Genital Symptoms
35		15	Hypochondriasis
36		16	Loss of Weight
37		17	Insight
38			Total HRSD score
39	Depression severity (BDI)	Item: 1	Sadness
40		2	Pessimism
41		3	Past Failure
42		4	Loss of Pleasure
43		5	Guilty Feelings
44		6	Punishment Feelings
45		7	Self-Dislike
46		8	Self-Criticalness
47		9	Suicidal Thoughts of Wishes
48		10	Crying
49		11	Agitation
50		12	Loss of interest
51		13	Indecisiveness
52		14	Worthlessness
53		15	Loss of energy
54		16	Changes in Sleeping Pattern
55		17	Irritability
56		18	Changes in Appetite
57		19	Concentration Difficulty
58		20	Tiredness or Fatigue
59		21	Loss of Interest in Sex
60			Total BDI score
**Depression subtypes, symptoms, and dimensions**			
61	Subtypes (SCAN)	1	Melancholic subtype
62		2	Atypical subtype
63		3	Anxious depression
64		4	Anxious-somatizing depression
65	Symptoms (SCAN)	Item: 1	Depressed mood

66		2	Anhedonia
67		3	Hopelessness
68		4	Loss of reactivity
69		5	Loss of interest
70		6	Inefficient thinking
71		7	Retardation
72		8	Loss of energy
73		9	Loss of libido
74		10	Loss of self-esteem
75		11	General anxiety
76		12	Phobia
77		13	Free-floating anxiety
78		14	Anxiety with autonomic symptoms
79		15	Pathological guilt
80		16	Guilty ideas of reference
81		17	Restlessness
82		18	Irritability
83		19	Preoccupation with death
84		20	Fatiguability
85		21	Morning depression
86		22	Suicidality
87		23	Early waking
88		24	Loss of appetite
89		25	Hypersomnia
90		26	Increased appetite
91		27	Appetite
92		28	Anxiety A
93		29	Anxiety B
94		30	Anxiety
95		31	Guilt
96		32	Irritability
97	Melancholic symptom count		
98	Atypical symptom count		
99	Symptom factors<br>(published factors analysis)	Factor 1	Observed mood
100		Factor 2	Cognitive
101		Factor 3	Neurovegetative
102	Dimensions		

	(published factors analysis)	Dim. 1	Mood
103		Dim. 2	Anxiety
104		Dim. 3	Pessimism
105		Dim. 4	Interest-activity
106		Dim. 5	Sleep
107		Dim. 6	Appetite
**Stressful life events**			
108	Stressful Life Events (LTE-Q)	Item: 1	Illness, injury or assault (Personal)
109		2	Illness, injury or assault (To a close relative)
110		3	Death of first degree relative
111		4	Death of second degree relative
112		5	Break off relationship
113		6	Serious problems with a close relative
114		7	Redundant or sacked Serious from job
115		8	Seeking work for more than 1 month
116		9	Serious financial problems
117		10	Problems involving police
118		11	Something valued was lost/stolen
119		12	Birth to a child (personal or partner)
120	Any SLE (yes/no)		
121	Total number of SLEs		
**Antidepressant medication History**			
122	Previous trials of antidepressants (number)		
123	History of taking any antidepressants (yes/no)		
124	Previous trials of SSRI antidepressants (number)		
125	History of taking SSRI antidepressants (yes/no)		
126	Previous trials of tricyclic antidepressants (number)		
127	History of taking tricyclic antidepressants (yes/no)		
128	Previous trials of SNRI antidepressants (number)		

129	History of taking SNRI antidepressants (yes/no)	
130	Previous trials of IMAO antidepressants (number)	
131	History of taking IMAO antidepressants (yes/no)	
132	Taking benzodiazepine at time of recruitment (yes/no)	
133	Taking Z-hypnotics at time of recruitment (yes/no)	
134	History of taking mirtazapine (yes/no)	
135	Number of previous trials of other antidepressants (number)	
136	History of taking other antidepressants (yes/no)	
137	Taking SSRI antidepressant at time of recruitment (yes/no)	
138	Taking antidepressant at time of recruitment (yes/no)	
139	Taking tricyclic antidepressant at time of recruitment (yes/no)	

Table S2: Sample description

	Both antidepressants		Escitalopram		Nortriptyline	
Sample size	n=430		n=220		n=210	
	Mean	SD	Mean	SD	Mean	SD
Age (years)	42.23	11.43	41.71	11.41	42.77	11.46
Age at onset (years)	31.38	10.79	32.11	10.95	30.61	10.58
Body Mass Index (BMI)	25.65	4.81	25.49	4.32	25.81	5.27
Education (years)	6.9	5.28	6.45	4.99	7.38	5.53
Montgomery-Åsberg Depression Rating Scale (MADRS) score (baseline)	29.37	6.56	28.92	6.45	29.83	6.65
Hamilton Depression Rating Scale-17 item score (baseline)	24.3	5.99	24.11	5.73	24.51	6.26
Beck Depression Inventory score (baseline)	28.73	9.29	28.58	8.99	28.90	9.61
No. of Stressful Life events during the 6 months before baseline	0.6	0.49	0.6	0.49	0.6	0.49
	N	%	N	%	N	%
Female gender	277	64.41	140	63.64	137	65.24
Smoker at baseline (yes)	177	41.16	94	42.73	83	39.52
Children (yes)	296	68.84	150	68.18	146	69.52
Partner (yes)	250	58.14	128	58.18	122	58.10
Occupation (yes)	253	58.84	136	61.82	117	55.71
Schedules for Clinical Assessment in Neuropsychiatry:						
Melancholic depresion	61	14.19	31	14.09	30	14.29
Atypical depression	29	6.74	14	6.36	15	7.14
Anxious-somatizing depression	236	54.88	103	53.18	119	56.67
Anxious depression	198	46.05	117	46.82	95	45.24
Experiencing at least one Stressful Life Event during the 6 months before baseline	258	60	133	60.45	125	59.52
History of antidepressant treatment	209	48.6	97	44.09	112	53.33
History of SSRI antidepressant	135	31.4	65	29.55	70	33.33
History of Tricyclic antidepressant	79	18.37	35	15.91	44	20.95
History of Dual Action Antidepressant	40	9.30	22	10.00	18	8.57

Table S3: Information about markers included in models predicting remission and previously reported gene associations.

Gene	*Marker*	*Chr:Position*	*Antidepressant*	*MAF*	*Allele*	*Gene associations*
COL25A1 - Intron variant	rs140236958	4:108960195	both	0.32	A/T	Encodes the CLAC protein, which has been implicated in Alzheimer's disease (AD) pathogenesis (Forsell et al. 2010) and in the growth and decay of the brain. Associated with Comorbid Antisocial Personality Disorder and Substance Dependence (Li D, et. al. , 2012).
COL25A1 - Intron variant	rs17596971	4:108926947	both	0.27	A/G	Encodes the CLAC protein, which has been implicated in Alzheimer's disease (AD) pathogenesis (Forsell et al. 2010) Associated with Comorbid Antisocial Personality Disorder and Substance Dependence (Li D, et. al. , 2012).
MAF – Intron variant	rs3784925	16:79571686	both	0.21	C/T	c-Maf cooperates with Sox9 to activate the type II collagen gene (Huang et al. , 2002).
TBC1D8 - intron variant	rs7556762	2:101149460	both	0.17	G/T	Osteoporosis (Hsu et al. , 2010) and pancreatic cancer (Pezzilli et al. , 2011) Predictive of risk of postpartum depression. (Landsman et al. , 2017)
Intergenic	rs1474552	9:792192	both	0.5	C/T	
ITGB2 – Intron variant	rs62314575	21:44917375	both	0.22	A/G	ITGB2 associated with deficits in synaptic connectivity along with several behavioural impairments (Zhan et al. ,2014, Stevens et al. ,2007, Schafer et al. ,2012, Paolicelli RC et al. ,2011, Kettenmann et al. ,2013). Associated to other several conditions: papillary thyroid cancer (Eun et al. , 2013), inflammatory mechanisms (Koch et al. , 2001), vasculitis (Meller et al. , 2001), Hirschsprung's disease (Moore et al. , 2008), repair of the infarcted myocardium (Wu et al. , 2006), obesity (Awaya et al. , 2008) and alcohol response (Joslyn et al. , 2010).
VTI1B – Intron variant	rs142319521	14:67675577	both	0.19	-/TA	Vesicle transport through interaction with t-SNAREs 1B
Intergenic	rs2400304	5: 101130050	both	0.21	C/T	
SERP1 – Intron variant	rs6794400	3:150581092	Nortriptyline	0.057	A/C	Stress-Associated Endoplasmic Reticulum Protein
TMEM170A – Intron variant	rs37596	16:75464422	Nortriptyline	0.32	A/C	Associated with coronary risk disease (Gertow et al. , 2012)
CFDP1 – Intron variant	rs8053632	16:75331042	Nortriptyline	0.23	C/T	Associated with coronary risk disease (Gertow et al. , 2012) and lung function (Soler Artigas et al. ,2011)
CCDC7 – Intron variant	rs111685823	10:32799271	Nortriptyline	0.0096	C/T	
TMEM2 – Intron variant	rs17057129	9:71698513	Nortriptyline	0.20	A/C	Regulator of heart development during myocardial and endocardial morphogenesis (Totong et al, 2011).
SGCZ – Intron variant	rs5889536	8:14517210	Nortriptyline	0.068	-/G	Part of the sarcoglycan complex, a group of six proteins which bridge the inner cytoskeleton and the extra-cellular matrix. Recently associated with major depression, schizophrenia and bipolar disorder (Chen et al., 2016). It has also been associated with alcohol and nicotine co-dependence (Zuo et al. 2012), and Parkinson's disease (Liu X, et al 2013).
SLC25A37 – Intron variant	rs34841556	8:23556091	Nortriptyline	0.446	-/CT	Its consistent down-regulation in MDD patients in three independent samples suggested that SCL25A37 may be used as a potential biomarker for MDD diagnosis (Huo YX et al. 2016). Evidence for association with fatigue (Hsiao et al. 2014)
ACCN1 - Intron variant	rs8082631	17:34064031	Nortriptyline	0.42	A/G	Evidence for association with response to lithium treatment in BD (Squassina et al. ,2011) and risk to autism (Stone et al. ,2007)
Intergenic	rs4773117	13:110066456	Nortriptyline	0.017	C/T	
Intergenic	rs79693177	2:186199515	Nortriptyline	0.026	G/T	
Intergenic	rs12874087	13:68211573	Nortriptyline	0.20	C/T	
Intergenic	rs2345113	14:56675149	Nortriptyline	0.15	C/G/T	
Intergenic	rs17091959	14:56691048	Nortriptyline	0.15	C/T	Between RPL36AP1 and OTX2 (OTX2 plays a role in brain and sensory organ development)
Intergenic	rs10792321	11:61979317	Nortriptyline	0.40	A/G	
Intergenic	rs199561596	2:186510855	Nortriptyline		-/AT	
Intergenic	rs144829540	2:186464172	Nortriptyline	0.15	A/G	

Intergenic	rs149619279	9:122105909	Nortriptyline	0.08	A/G	
Intergenic	rs34319049	20:38710108	Nortriptyline	0.03	C/T	
Intergenic	rs151132095	2:186317904	Nortriptyline	0.15	C/T	
Intergenic	rs4279984	11:37172240	Nortriptyline	0.094	C/T	
TMEM229B	rs28373080	14:67506046	Escitalopram	0.49	C/T	Evidence for association with risk for Parkinson disease (Nalls et al. ,2014) Evidence of association with childhood obesity in the Hispanic population (Comuzzie et al, 2012)
CDYL – Intron variant	rs7757702	6:4940209	Escitalopram	0.45	A/T	
LOC105375673 – Intron variant	rs2704022	8:100728509	Escitalopram	0.42	A/C	
Intergenic	rs1891943	13:53013037	Escitalopram	0.13	A/G	
Intergenic	rs151139256	2:180139767	Escitalopram	0.026	-/T	
Intergenic	rs11002001	10:52426412	Escitalopram	0.014	A/G	
Intergenic	rs62182022	2:180060581	Escitalopram	0.15	C/T	
Intergenic	rs76557116	13:100011900	Escitalopram	0.47	C/T	
Intergenic	rs9557363	13:100032511	Escitalopram	0.47	C/T	
Intergenic	rs1392611	4:45347307	Escitalopram	0.16	C/T	
Intergenic	rs10812099	9:24797940	Escitalopram	0.23	A/T	

Table S4: Variables selected and Odds ratio from elastic net logistic regression models estimated in the training data sets for the whole set of individuals, either treated with escitalopram or nortriptyline.

Both antidepressants<br>N train=280; N test=150	
**Predictor**	**OR**
Appetite	0.89
HRSD total	0.91
Observed mood	0.93
rs142319521	0.85
rs1474552	0.9
rs10976609	0.92
rs7556762	0.93
rs3784925	0.95
rs62314575	0.95
rs17596971	0.97
rs2400304	0.99
rs140236958	0.99
